# Intestinal decompression and drainage in preventing post-endoscopic submucosal dissection electrocoagulation syndrome in colorectal ESD: a prospective study

**DOI:** 10.1093/gastro/goaf020

**Published:** 2025-04-15

**Authors:** Yunpeng Dong, Jiao Liu, Wen Jia, Meng Zhang, Xuezhu Wang, Meiling Lin, Zhuo Yang

**Affiliations:** Department of Digestive Endoscopy, General Hospital of Northern Theater Command, Shenyang, Liaoning, P. R. China; Department of Digestive Endoscopy, General Hospital of Northern Theater Command, Shenyang, Liaoning, P. R. China; Department of Digestive Endoscopy, General Hospital of Northern Theater Command, Shenyang, Liaoning, P. R. China; Department of Digestive Endoscopy, General Hospital of Northern Theater Command, Shenyang, Liaoning, P. R. China; Department of Digestive Endoscopy, General Hospital of Northern Theater Command, Shenyang, Liaoning, P. R. China; Department of Digestive Endoscopy, General Hospital of Northern Theater Command, Shenyang, Liaoning, P. R. China; Department of Digestive Endoscopy, General Hospital of Northern Theater Command, Shenyang, Liaoning, P. R. China

**Keywords:** colorectal cancer, colorectal ESD, intestinal decompression drainage tube, electrocoagulation syndrome

## Abstract

**Background and aims:**

This study explored the efficacy of a prophylactic intestinal decompression tube in reducing the incidence of post-endoscopic submucosal dissection electrocoagulation syndrome (PECS).

**Methods:**

A total of 157 eligible patients with colorectal mucosal lesions scheduled for endoscopic submucosal dissection (ESD) were prospectively recruited; after drop out 11 patients, 146 patients were randomly assigned to an experimental group (group 1, *n *=* *73) or control group (group 2, *n *=* *73). Patients in the experimental group underwent placement of an intestinal decompression drainage tube after ESD, while the control group received no additional treatment after ESD. The primary outcome was the incidence of PECS. Secondary outcomes included the incidence of postoperative complications, time to removal of the intestinal decompression tube, the degree of abdominal pain as measured by the visual analog scale (VAS), and the participants’ self-rated comfort level with the intestinal decompression tube.

**Results:**

A total of 146 patients (*n *=* *73 per group) were finally analyzed between July 2022 and February 2023. All tumors were successfully resected en bloc. A significant difference in the incidence of PECS was found between group 1 and group 2 (5.5% vs 16.4%; *P *=* *0.034). Precisely, 61.6% of patients felt painless for intestinal decompression tube, and no severe or unbearable pain was reported.

**Conclusions:**

The placement of intestinal decompression drainage tube could reduce the incidence of PECS after colorectal ESD, which might play a preventive role in the occurrence of PECS.

## Introduction

Over the past decade, colorectal cancer (CRC) incidence and mortality rates have consistently increased worldwide [1]. Minimally invasive endoscopic treatment techniques have been utilized to cure early-stage CRC, with endoscopic submucosal dissection (ESD) representing the mainstay of treatment, especially after significant inroads in ESD techniques in recent years [2–6]. Resection of colorectal mucosal lesions can pose a challenge, and the risk of postoperative complications is increased due to the rich vasculature and thinness of the colorectal wall, as well as the presence of large curvatures and folds [7, 8]. It is widely acknowledged that the adverse events after ESD include bleeding, perforation, and post-ESD electrocoagulation syndrome (PECS). The incidence rates of postoperative perforation and bleeding are estimated to be 2%–14% and 0.7%–3.1%, respectively [9–12], while PECS occurs in 4.8% to 40% of patients [13–18]. If PECS occurs clinically, it will lead to fever, abdominal pain, and other symptoms in patients, which will prolong the hospital stay of patients and bring unnecessary physical, economic, and even mental burden to patients. At present, the understanding of PECS after colorectal ESD is still unclear, which requires increased attention and further exploration.

No consensus has been reached on the definition of PECS, although it has been established that it typically presents with peritoneal inflammation (such as fever, leukocytosis, tenderness, and rebound tenderness) in the absence of perforation [15]. The specific pathological mechanism of PECS remains unclear. There are two main hypotheses: one suggests that inflammation of the muscularis propria caused by excessive coagulation during ESD leads to PECS, while the other suggests that the inflammatory reaction is caused by an intestinal infection due to mucosal defects after ESD [14, 19].

Building upon findings reported in the literature, we hypothesized that intestinal decompression could be an effective preventive measure against PECS following colorectal ESD. We put this hypothesis to the test by comparing the occurrence rates of PECS and other complications in patients who received an intestinal decompression drainage tube and those who did not.

## Materials and methods

### Study design

The institutional review board of the General Hospital of Northern Theater Command approved this single-center, prospective, randomized trial [approval number Y(2022)068], and this study was registered in the Chinese Clinical Trial Registry (ChiCTR220006381). After comprehensively explaining the study’s goals, all eligible participants or their families gave informed consent. The study adhered to the Declaration of Helsinki (KUH1010556).

Patients with colorectal mucosal lesions scheduled for ESD in General Hospital of Northern Theater Command (Liaoning, P. R. China) between July 2022 and February 2023 were included and randomly divided into an experimental group or a control group with a ratio of 1:1 according to identification numbers generated by Microsoft Excel 2018 (Microsoft Corporation, Redmond, Washington, USA). In the experimental group, an intestinal decompression drainage tube was placed after ESD. All patients were evaluated by preoperative endoscopic ultrasonography or magnifying colonoscopy and were included according to the indications of colorectal ESD identified in the Japan Gastroenterological Endoscopy Society guidelines (2020) [20].

The inclusion criteria were as follows: (i) patients aged ≥18 years; (ii) patients had indications for colorectal ESD; (iii) patients with normal coagulation function; and (iv) voluntary participation in the study.

The exclusion criteria were as follows: (i) pregnant or lactating women, as well as those who may become pregnant or are preparing for pregnancy during the clinical trial period; (ii) patients with other severe organic diseases that may affect the evaluation of this study, such as severe liver disease, heart disease, kidney disease, etc.; (iii) patients who have discontinued antiplatelet and non-steroidal anti-inflammatory drugs for less than 7 days; (iv) patients who lack autonomy or cannot accurately express their complaints, such as mental illness, severe neurosis, or those who cannot cooperate with this trial; (v) patients with submucosal tumors in the colon; (vi) patients participating in other clinical studies that may affect the observation of the results of this study simultaneously; (vii) subjects with loss of clinical data or inability to effectively cooperate with follow-up; or (viii) other situations deemed inappropriate for inclusion by researchers.

**Figure 1.**
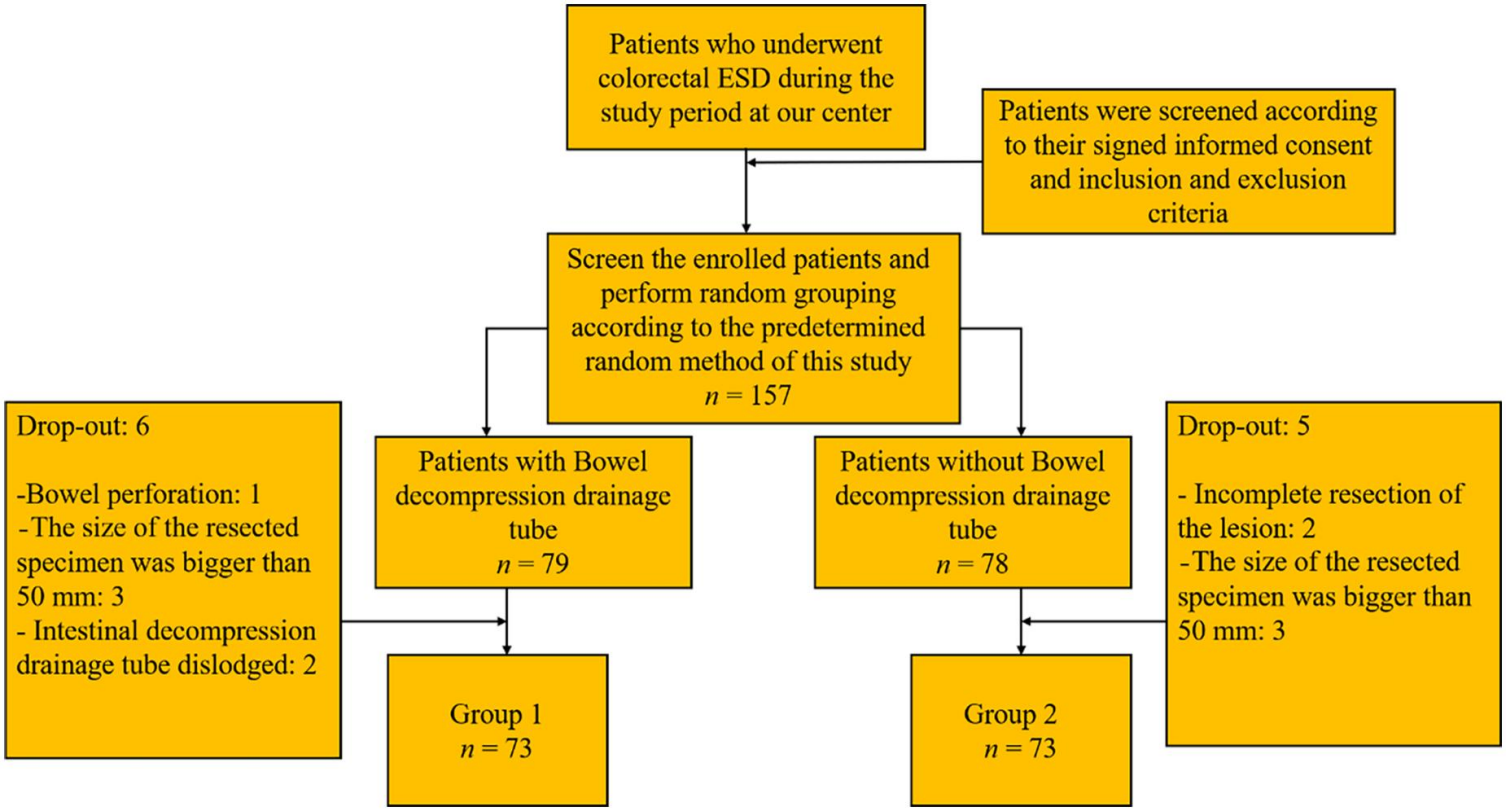
Flowchart of the study. A total of 157 patients who underwent colorectal endoscopic submucosal dissection were randomly assigned to 1 of 2. After excluding two cases of incomplete resection of the lesion, one case of intraoperative perforation, six cases of lesion with a maximum diameter >5 cm, and two cases of postoperative decompression tube detachment, 146 patients were finally analyzed. ESD = endoscopic submucosal dissection.

The criteria for withdrawal from the trial were as follows: (i) patients who stop surgery midway or have incomplete resection of lesions; (ii) specimens with a maximum diameter of >5 cm after resection; (iii) intestinal perforation during or after surgery; (iv) patients undergoing multiple ESD surgeries simultaneously; (v) detachment of the decompression tube before the end of the trial; (vi) patients experiencing severe complications during the trial and cannot tolerate them; (vii) patients developing other diseases during treatment that may interfere with the observation of this trial; or (viii) patients lost to follow-up (Figure 1).

### Intestinal decompression drainage tube

**Figure 2.**
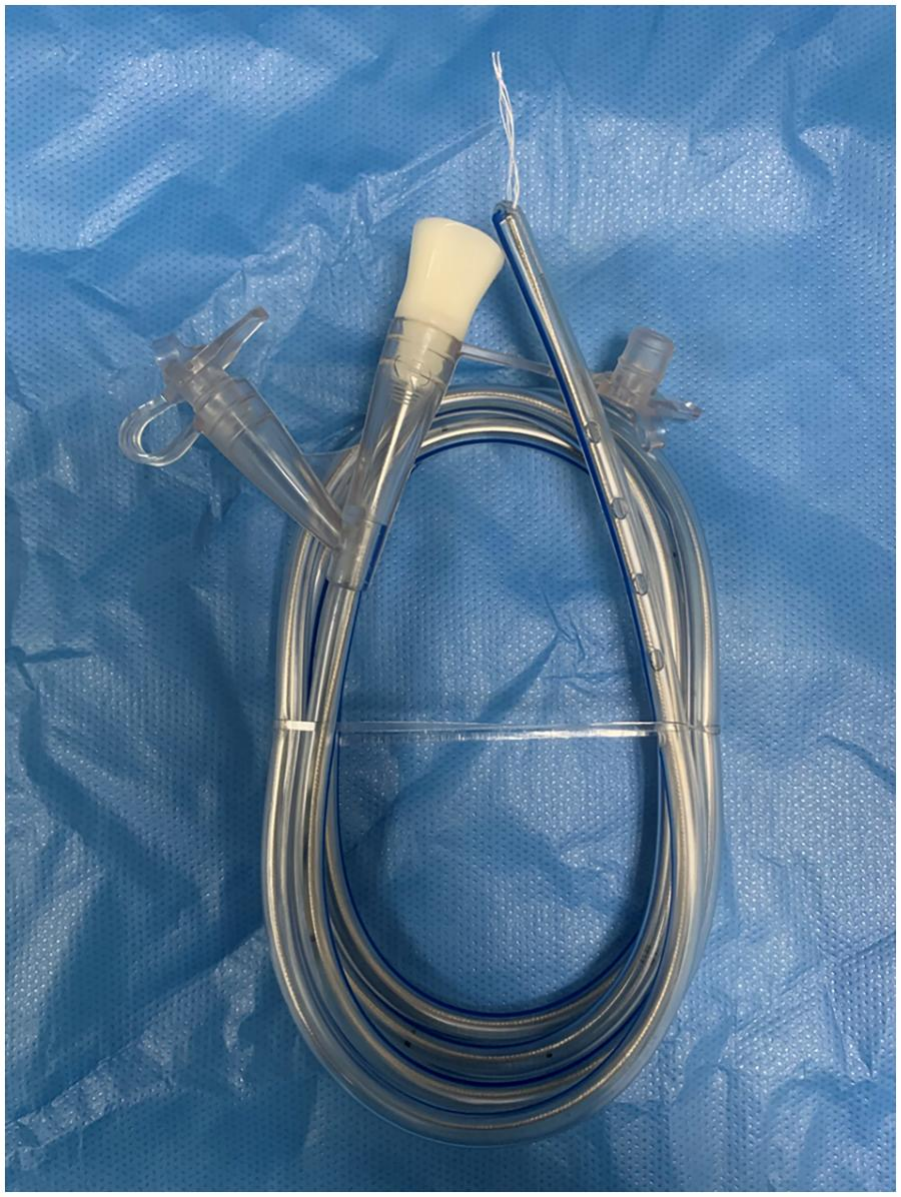
Intestinal decompression drainage tube.

For this study, we developed an intestinal decompression drainage tube with a side hole that enables drainage and is made of soft silicone material that provides support. The proximal end of the drainage tube is attached to a cotton thread, while the distal end is connected to a drainage bag. During use, the decompression drainage tube is secured to the wound intestinal mucosa with a metal entrainment tube, the tip of the tube is not inserted at the site of the ESD ulcer and the side hole is positioned parallel to the wound surface. The distal end of the tube is affixed to the skin around the buttocks with adhesive tape and connected externally to a drainage bag (Figure 2).

### Observation indicators

Locations of colorectal lesions determined by the Japanese Society for Cancer of the Colon and Rectum (JSCCR) [21] include ileocecal, ascending colon, transverse colon, descending colon, sigmoid colon, and rectum. The gross appearance of lesions was based on the Paris-Japanese classification and the Kudo classification system [22], including protuberant tumors (type 0-I) or laterally spreading tumor (LST). LST was defined as lesions greater than 1 cm with low height extending laterally along the lumen wall and could be classified into granular (LST-G) and nongranular (LST-NG). The size of the resected specimen was determined by the maximum diameter of the specimen after the specimen was stretched on a scale plate and pinned. Lesion specimens were then sent to the Department of Pathology for evaluation by an experienced pathologist. Histological diagnosis was based on the World Health Organization classification system of adenomas (tubular + villous + tubulovillous), adenomas with high-grade dysplasia, carcinomas, and others. Complete resection referred to the absence of tumor cells at the lateral and basal margins. ESD operation time was defined as the time from circumferential marking to complete lesion removal. ESD failure was defined as failure to complete the resection procedure and forced discontinuation.

PECS was defined as transient tenderness and rebound tenderness complicated with fever (≥37.8 °C), leukocytosis (≥10,800 cells/μL), or elevated C-reactive protein without perforation after ESD. Perforation was ruled out, with no free abdominal or subphrenic air observed on postoperative imaging.

Intraoperative perforation refers to macroscopic perforation caused by improper operation. Microscopic perforation indicated postoperative intestinal perforation [8] with free air confirmed by abdominal radiography or CT scan. Delayed perforation was established based on evidence of perforation after completion of the ESD procedure.

Bleeding after ESD was defined as ESD-related bleeding with hematochezia, melena, hypotension, or decreased hemoglobin levels. Major bleeding was defined as bleeding requiring blood transfusion or endoscopic intervention [16].

**Figure 3.**
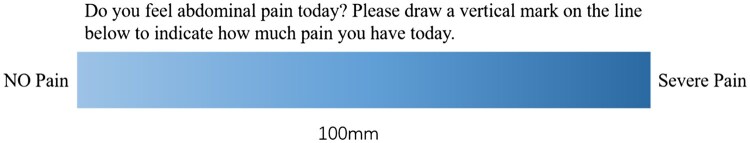
Visual analog scale.

A visual analog scale (VAS) was used to evaluate the degree of abdominal pain (0Z = no pain; 10Z = maximum pain). VAS is a simple descriptive pain scale that consists of a horizontal line with a length of 100 mm, where 0 mm represents no pain and 100 mm represents the most severe pain. Abdominal pain was defined when the VAS score exceeded 30 mm (patients marked the severity of his/her pain on the line from 0 mm) (Figure 3).

Comfort level of intestinal decompression tube was a self-rating subjective comfort evaluation using numerical rating scale (NRS). Patients were asked to express the severity of pain with numbers 1 to 10. Scores of 0, 1–3, 4–6, and 7–10 indicated no pain, mild pain (pain did not affect sleep), moderate pain, and severe pain (inability to fall asleep or wake up from sleep), respectively.

### Sample size calculation

Based on the literature, the incidence of PECS ranges from 4.8% to 40% [13–18]. A pilot study was conducted to determine the necessary sample size. The pilot study involved 40 patients subsequently included in the current study. The experimental and control groups had a 1:1 ratio, and the incidence of PECS was 5% and 20%, respectively. One-sided testing was employed with a significance level of 5% (α = 0.05) and a test power of 80% (β = 0.2). PASS 15.0 software was used to calculate the sample size, which resulted in N1 = N2 = 58 cases in each group. Given that 20% of participants were expected to drop out of the study, 146 eligible subjects were finally included and randomly allocated into two groups (*n *=* *73 each).

### Surgical procedures and standards

**Figure 4.**
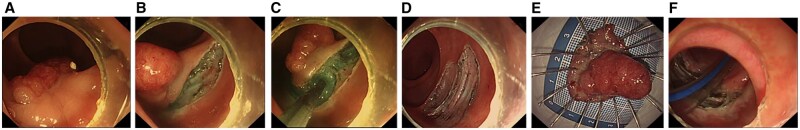
The specific procedures of endoscopic submucosal dissection. (A) Marking. (B) Injection. (C) Incision. (D) Stripping. (E) and (F) Postoperative catheterization and pathological examination.

All ESD procedures were performed by three experienced endoscopists, each of whom had completed over 100 ESD procedures. Patients were given a liquid diet 3 days before the operation, and cellulose-rich foods in were avoided. Patients orally took two bags of compound polyethylene glycol electrolyte powder and 2,000 mL of water 6 h before the operation for intestinal cleaning. Under the supervision of experienced anesthesiologists, patients were given intravenous midazolam and propofol for moderate sedation. The endoscope utilized in the procedure was a commonly employed visual endoscope manufactured by Olympus Corporation (GIF-HQ290, GIF-Q260J; Tokyo, Japan) and equipped with a disposable distal attachment (D-201–11804; Olympus Corporation). Carbon dioxide was utilized for endoscopic insufflation during the procedure. Other surgical equipment used included a 200D system ERBE (Erbe Elektromedizin GmbH, Tübingen, Germany) high-frequency generator, multi-functional knife (MFK, AMH-EK-O-2.4X1800(4)-N, Anrei, China), disposable injection needle (NM-4L-1; Olympus; Tokyo, Japan), disposable Hook snare (ASM-1- S, Wilson-Cook Medical Inc, USA) hot biopsy forceps (FD-410LR; Olympus; Tokyo, Japan), disposable metal hemostatic clip (ROCC-D-26–195, Micro-Tech, Nanjing, China) 0.9% sodium chloride injection, methylene blue, and sodium hyaluronate (Figure 4).

### Perioperative management and follow-up

All patients were hospitalized at least 1 day before ESD and underwent vital sign measurements, physical examination, radiographic examination (i.e. chest and abdominal examinations), and blood tests.

Patients underwent fasting, liquid fasting and daily fluid infusion for 1 to 3 days after the ESD procedure. The transition to a liquid diet was gradually initiated based on the patient’s condition. Meanwhile, antibiotics and hemostatic drugs were used according to the actual situation, abdominal pain and changes in abdominal signs were recorded, and if necessary, an abdominal X-ray or CT examination was conducted to detect delayed perforation. In the experimental group, anal canal prolapse was observed and recorded daily. Tetracaine gel was applied to the perianal area for lubrication, and local nursing was performed if necessary. Nursing staff measured and recorded body temperature twice daily at 8:00 a.m. and 20:00 p.m., which could be increased based on the doctor’s discretion. If the patient experienced fever, their blood routine and C-reactive protein were promptly reexamined. The attending physician assessed the level of abdominal pain using the VAS daily after the operation.

Postoperative extubation time could be adjusted according to the drainage condition and the patient’s recovery condition, and the time was recorded. The decompression drainage tube was generally retained for at least 72 h until the patient returned to a normal diet.

Three follow-up visits were scheduled in this study: intraoperatively and on postoperative day 1 (POD1) and POD3. Complications, including PECS, were identified by monitoring the patients’ body temperature, inflammatory indicators, and VAS. Furthermore, in the experimental group, the status of the intestinal decompression drainage tube was recorded, which included self-reported comfort level, extubation, and the time of extubation.

### Statistical analysis

Variables were expressed as mean ± standard deviation, and differences between the two groups were assessed by *t*-test. Categorical variables were expressed as counts (proportion), and the chi-square test was used for group comparisons. All results were recorded to units or two decimal places. The threshold for significance was set at *P *<* *0.05. All statistical analyses were conducted using SPSS, Version 25.0 (SPSS Inc., Chicago, IL, USA).

## Results

### Demographics and resected lesions at baseline

A total of 157 patients were initially included between June 2022 and February 2023. Following the exclusion of cases that involved incomplete lesion resection (*n *=* *2), intraoperative perforation (*n *=* *1), lesions with a maximum diameter exceeding 5 cm (*n *=* *6), and postoperative decompression tube detachment (*n *=* *2), the final analysis included 146 patients.

Baseline characteristics are shown in Table 1. The mean age of group 1 was 57.5 ± 12.2 years with 37.0% female, and the mean age of group 2 was 58.6 ± 10.9 years with 35.6% female. The mean maximum diameter of the lesion was 2.41 ± 0.83 cm and 2.41 ± 0.85 cm in group 1 and group 2, respectively. Besides, the most common lesion location in both groups was the rectum, including 34 (46.6%) cases in group 1 and 22 cases (30.1%) in group 2. LST-G was the most frequent appearance (45.2%) of lesion in group 1, while type 0–I was the most frequent appearance (57.5%) of lesion in group 2. Adenomas accounted for 53.4% and 52.1% in group 1 and group 2, respectively. No significant differences were found between the two groups in age, gender, size of lesion, location of lesion, gross appearance of lesion, and morphology.

**Table 1.** Baseline characteristics of patients and resected tumors

Variable	Group 1 (*n *=* *73)	Group 2 (*n *=* *73)	*P* value
Female, *n* (%)	27 (37.0)	26 (35.6)	0.863
Age, years, mean ± SD	57.5 ± 12.2	58.6 ± 10.9	0.570
Tumor size, mm, mean ± SD	2.41 ± 0.83	2.41 ± 0.85	0.984
Tumor location, *n* (%)			0.110
Rectum	34 (46.6)	22 (30.1)	
Sigmoid colon	11 (15.1)	16 (21.9)	
Descending colon	5 (6.8)	2 (2.7)	
Transverse colon	4 (5.5)	12 (16.4)	
Ascending colon	10 (13.7)	10 (13.7)	
Cecum	9 (12.3)	11 (15.1)	
Tumor morphology, *n* (%)			0.254
0–I	32 (43.8)	42 (57.5)	
LST-G	33 (45.2)	25 (34.2)	
LST-NG	8 (11.0)	6 (8.2)	
Histologic type, *n* (%)			0.832
Adenoma	39 (53.4)	38 (52.1)	
High-grade dysplasia	16 (21.9)	17 (23.3)	
Adenocarcinoma	15 (20.5)	17 (23.3)	
Others	3 (4.1)	1 (1.4)	

0–I = protuberant tumors, LST-G = laterally spreading tumor-granular, LST-NG = laterally spreading tumor-nongranular, SD = standard deviation.

### Intraoperative and postoperative primary outcomes

Table 2 displays the intraoperative and postoperative indicators. The incidence of PECS was significantly lower in group 1 than in group 2 (5.5% vs 16.4%, *P *=* *0.034). According to our findings, the drainage tube successfully achieved the desired drainage effect in group 1, and the mean extubation time after surgery was 3.5 ± 0.7 days. Regarding the comfort level of the intestinal decompression tube, 61.6% of patients reported no pain, and no patients reported severe or unbearable pain. There were no significant differences in operation time, wound closure, delayed bleeding, or postoperative abdominal pain between the two groups. No patient experienced delayed perforation. Moreover, no significant differences were found between the two groups regarding the highest postoperative body temperature, C-reactive protein, and leukocyte count. Additionally, the duration of hospital stay was comparable between groups 1 and 2 (8.0 ± 1.5 vs 7.5 ± 1.8 days, *P *=* *0.198). There were no significant differences in hospital cost between the two groups (2,933.3 ± 648.9 vs 2,799.5 ± 575.9 USD, *P *=* *0.190).

**Table 2.** Comparison of endoscopic data and outcomes between group 1 and group 2

Variable	Group 1 (*n *=* *73)	Group 2 (*n *=* *73)	*P* value
Procedure time, min, mean ± SD	49.49 ± 14.27	47.84 ± 10.97	0.433
Post-ESD wound closure, *n* (%)	26 (35.6)	34 (46.6)	0.852
Post-ESD bleeding, *n* (%)	1 (1.4)	2 (2.7)	1.000
Post-ESD perforation, *n* (%)	0 (0)	0 (0)	1.000
VAS >30 mm, *n* (%)	9 (12.3)	8 (11.0)	0.796
Max body temperature during hospitalization, °C, mean ± SD	36.91 ± 0.42	37.06 ± 0.57	0.071
Post-ESD maximum CRP, mg/dL	1.80 (1.28–4.05)	1.86 (1.20–5.45)	0.688
Day after ESD leukocyte count, 10^3^/mm^3^	5.48 ± 10.37	5.62 ± 8.88	0.928
Total hospital stay, days, mean ± SD	8.0 ± 1.5	7.5 ± 1.8	0.198
Total hospital cost, USD, mean ± SD	2933.3 ± 648.9	2799.5 ± 575.9	0.190
PECS, *n* (%)	4 (5.5)	12 (16.4)	0.034
Post-ESD extubation time, days, mean ± SD	3.5 ± 0.7	–	–
NRS, *n* (%)			
Painless	45 (61.6)	–	–
Mild pain	25 (34.2)	–	–
Moderate pain	3 (4.1)	–	–
Severe pain	0 (0)	–	–

SD = standard deviation, ESD = endoscopic submucosal dissection, VAS = visual analog scale, CRP = C-reactive protein, NRS = numerical rating scale.

### Incidence of PECS

Table 3 presents the comparison of demographics and clinical data between patients who developed PECS and those who did not. Of the 146 patients, 16 were diagnosed with PECS (4 in the experimental group and 12 in the control group), while the remaining 130 did not exhibit any signs of PECS. The PECS group comprised 4 females and 12 males with a mean age of 57.8 ± 14.5 years. The most common lesion location in the PECS group was the rectum (37.5%), with type 0–I being the most common lesion (56.3%), and adenomas being the prevalent histological type (56.3%). The lesion size was significantly larger in patients with PECS than those without (3.03 ± 1.10 vs 2.35 ± 0.79 cm, *P *=* *0.002). The PECS group was associated with a significantly longer operation time than the non-PECS group (54.9 ± 10.2 vs 47.9 ± 12.8 min, *P *=* *0.038). Although wound closure could reduce wound contamination and prevent PECS, the difference between the two groups was not statistically significant (*P *=* *0.480). Notably, the incidence of PECS was lower in patients with an intestinal decompression drainage tube (*P *=* *0.034). Compared with the non-PECS group, the PECS group was associated with a significantly longer hospital stay (10.1 ± 2.0 vs 7.6 ± 1.5 days, *P *<* *0.001).

**Table 3.** Comparison of demographics and clinical data between patients who developed PECS and those who did not

Variable	PECS (*n *=* *16)	Non-PECS (*n *=* *130)	*P* value
Female, *n* (%)	4 (25.0)	49 (37.7)	0.319
Age, years, mean ± SD	57.8 ± 14.5	58.08 ± 11.26	0.930
Tumor size, mm, mean ± SD	3.03 ± 1.10	2.35 ± 0.79	0.002
Tumor location, *n* (%)			0.497
Rectum	6 (37.5)	50 (38.5)	
Sigmoid colon	1 (6.3)	26 (20.0)	
Descending colon	0 (0)	7 (5.4)	
Transverse colon	2 (12.5)	14 (10.8)	
Ascending colon	4 (25.0)	16 (12.3)	
Cecum	3 (18.8)	17 (13.1)	
Tumor morphology, *n* (%)			0.743
0–I	9 (56.3)	65 (50.0)	
LST-G	5 (31.3)	53 (40.8)	
LST-NG	2 (12.5)	12 (9.2)	
Histologic type, *n* (%)			0.750
Adenoma	9 (56.3)	69 (53.1)	
High-grade dysplasia	2 (12.5)	29 (22.3)	
Adenocarcinoma	5 (31.3)	28 (21.5)	
Others	0 (0)	4 (3.1)	
Procedure time, min, mean ± SD	54.9 ± 10.2	47.9 ± 12.8	0.038
Procedure time >60 min, *n* (%)	7 (43.8)	22 (16.9)	0.027
Post-ESD wound closure, *n* (%)	8 (50.0)	53 (40.8)	0.480
Intestinal decompression drainage tube, *n* (%)	4 (25.0)	69 (53.1)	0.034
Total hospital stay, days, mean ± SD	10.1 ± 2.0	7.6 ± 1.5	<0.001

PECS = post-endoscopic submucosal dissection electrocoagulation syndrome, SD = standard deviation, 0–I = protuberant tumors, LST-G = laterally spreading tumor-granular, LST-NG = laterally spreading tumor-nongranular, ESD = endoscopic submucosal dissection.

## Discussion

This prospective study investigated the effect of intestinal decompression and drainage on the incidence of PECS among patients with colorectal mucosal lesions. Our study suggests that the prophylactic placement of an intestinal decompression drainage tube might be a protective factor against PECS.

The post-polypectomy coagulation syndrome was first introduced by Waye in 1981 [23], originally referring to abdominal pain, fever, leukocytosis, and peritonitis after endoscopic resection in the absence of obvious perforation. Although the incidence of post-polypectomy coagulation syndrome is reportedly 1% [1], there has been a rise in reported cases of PECS. However, with advancements in ESD techniques, the incidence of PECS has decreased. According to the literature, the PECS incidence rates range from 4.8% to 40% [13–18]. In contrast, our study found that the incidence of PECS was 5.5% in the experimental group and 16.4% in the control group.

Intestinal decompression and drainage are frequently used in the palliative treatment of surgical diseases, such as transanal tube placement after rectal cancer surgery to prevent anastomotic leakage [24] and endoscopic decompression with a transanal drainage tube to treat acute bowel obstruction [25]. However, little is currently known about preventing PECS after colorectal surgery. The possible functions of the drainage tube are as follows: first, it helps to discharge residual feces and gas and keeps the anal sphincter open, thereby reducing the pressure in the intestinal lumen; second, it reduces the chance of wound contamination by helping to discharge residual feces; third, by observing the color and properties of the extracted fluid, the postoperative wound condition can be monitored. Under normal circumstances, the drained fluid is a yellow intestinal secretion, but if a dark-colored fluid is continuously drained, clinicians should be vigilant for delayed wound bleeding and infection.

Moreover, there are potential adverse effects associated with placing an intestinal decompression drainage tube, such as spontaneous tube dropping and discomfort of the perianal skin. A previous study documented intestinal perforation following prophylactic placement of an anal decompression tube to prevent anastomotic leakage, but no such cases were observed in this study. Two patients withdrew from the trial due to the spontaneous dropping of the decompression tube, which might be related to improper fixation during placement or a change in body position during later care. During the self-rated evaluation of patient comfort level with the decompression tube, 61.6% of patients reported no pain, and 34.2% patients reported mild pain, indicating that most patients could tolerate the discomfort caused by the decompression tube. Warm water sitz baths or Vaseline and other lubricants around the anus could alleviate perianal discomfort. If necessary, tetracaine gel could be applied for pain relief. The timing of drain removal is also an important consideration. Consistent with previous studies that suggest a minimum of 3 days for indwelling drainage tubes, the average extubation time in our study was 3.5 ± 0.7 days. Tube extubation should be gentle, and local infiltration drugs, such as tetracaine gel, should be applied around the anus before slowly removing the tube.

Upon multivariate logistic regression analysis of risk factors for PECS, procedure time ≥60 min was an independent risk factor for PECS (odds ratio [OR] 4.390; 95% confidence intervals [95% CI] 1.221–15.790; *P *=* *0.023). Regrettably, we did not observe a correlation between lesion size and PECS, possibly due to the limited sample size. We intend to increase the sample size for future research endeavors. Therefore, we recommend implementing intestinal decompression drainage tubes for patients which procedure time ≥60 min.

The exact pathological mechanism behind PECS is not fully understood. In this study, we hypothesized that placing an intestinal decompression drainage tube may prevent PECS by reducing tension on the wound and promoting gas discharge in the intestinal cavity. By draining feces from the intestine, the tube lowers the risk of bacterial wound contamination, ultimately reducing the incidence of PECS.

It is now understood that thermal injury-related factors contribute to PECS, with high Joule heat increasing the risk of PECS. Our study further substantiated that the lesion size (*P *=* *0.002) and operation time (*P *=* *0.038) are associated with PECS. Larger tumors require more coagulation during dissection, leading to a longer operation time and increased Joule heat in the wound, making the stripped mucosa prone to an inflammatory reaction. Additionally, the sequence and time of electrocoagulation procedures vary among operators, and prolonged ESD can consistently lead to high Joule heat accumulation, causing serious damage to the stripped mucosal surface and increasing the risk of PECS. Experienced physicians spend less time on electrocoagulation procedures, resulting in shorter operation times, which can reduce the incidence of PECS.

Preoperative diet management and intestinal cleaning are crucial factors to consider. In this study, all patients achieved the qualified standard for intestinal cleansing. Jalanka *et al.* [26] demonstrated that adequate bowel cleansing prior to colonoscopy reduced the total microbiota to around 34.7 times the total microbiota in normal stool samples at baseline. During ESD, a large area of mucosal defect is created by surgery, leading to the contact of damaged mucosa with numerous intestinal bacteria and resulting in inflammation. Studies have isolated coagulase-negative staphylococci, *Bacillus subtilis*, and other bacilli from blood after ESD surgery, though the likelihood of bacteremia is low [27]. Kato *et al.* [28] identified ESD-related endotoxemia as one of the risk factors for ESD-related inflammatory response. While prophylactic antibiotic therapy is recommended for coagulation syndrome after cholecystectomy, its use in preventing PECS remains controversial. A prospective study found that prophylactic antibiotics administered after ESD surgery can prevent PECS and relieve local inflammatory symptoms [15]. However, another multicenter prospective study rejected this view and concluded that perioperative antibiotics are ineffective in reducing the incidence of PECS in patients undergoing colorectal ESD. Antibiotics may prevent infections caused by intestinal bacteria but cannot prevent all types of PECS caused by electrocautery-induced inflammation [29]. In this study, all patients did not receive antibiotic prophylactic treatment around the perioperative period, which prevented verification of the effect of prophylactic antibiotic use on preventing PECS.

The limitations of this study should be acknowledged. First, the sample size was relatively small, and the study design was limited to a single center. In the future, we plan to conduct a multicenter randomized controlled trial with a larger sample size to improve the generalizability of the findings. Besides, the abdominal pain VAS and the self-rating comfort level of the intestinal decompression tube are subject to certain subjectivity and individual differences. Moreover, despite all surgeons being experienced endoscopists, there may still be differences in their operating habits that cannot be fully standardized. In addition, this study did not specify the treatment modality for the wound surface, and each endoscopist had the discretion to decide whether to clip the mucosal defect after ESD. Finally, there was no in-depth investigation into the wound closure technique after ESD.

## Conclusions

In summary, the prophylactic placement of an intestinal decompression drainage tube may reduce the incidence of PECS after colorectal ESD without causing significant discomfort or postoperative complications. However, multicenter studies with larger sample sizes are warranted to increase the robustness of our findings.

## Authors’ contributions

Y.D., Z.Y., and J.L. were responsible for conception and design; Y.D., M.Z., and X.W. were responsible for analysis and interpretation of the data; Y.D. and M.L. were responsible for drafting of the article; Z.Y., J.L., and W.J. were responsible for critical revision for important intellectual content. All authors have read and approved the final version of the manuscript.

## Funding 

This work was supported by the key Research foundation of China [grant number 2017YFC0112303] and foundation of Science & Technology Department of Shenyang city [grant number 223213215].

## Conflicts of interest 

None declared.
